# Correlation-driven machine learning for accelerated reliability assessment of solder joints in electronics

**DOI:** 10.1038/s41598-020-71926-7

**Published:** 2020-09-09

**Authors:** Vahid Samavatian, Mahmud Fotuhi-Firuzabad, Majid Samavatian, Payman Dehghanian, Frede Blaabjerg

**Affiliations:** 1 Department of Electrical Engineering, Sharif University of Technology, 68260 Tehran, Iran; 2 Department of Advanced Materials and Renewable Energy, Iranian Research Organization for Science and Technology (IROST), 33535111 Tehran, Iran; 3 Department of Electrical and Computer Engineering, The George Washington University, Washington, DC 20052 USA; 4 Department of Energy Technology, Aalborg University, 9100 Aalborg, Denmark

**Keywords:** Energy science and technology, Engineering

## Abstract

The quantity and variety of parameters involved in the failure evolutions in solder joints under a thermo-mechanical process directs the reliability assessment of electronic devices to be frustratingly slow and expensive. To tackle this challenge, we develop a novel machine learning framework for reliability assessment of solder joints in electronic systems; we propose a correlation-driven neural network model that predicts the useful lifetime based on the materials properties, device configuration, and thermal cycling variations. The results indicate a high accuracy of the prediction model in the shortest possible time. A case study will evaluate the role of solder material and the joint thickness on the reliability of electronic devices; we will illustrate that the thermal cycling variations strongly determine the type of damage evolution, i.e., the creep or fatigue, during the operation. We will also demonstrate how an optimal selection of the solder thickness balances the damage types and considerably improves the useful lifetime. The established framework will set the stage for further exploration of electronic materials processing and offer a potential roadmap for new developments of such materials.

## Introduction

Massive development and widespread deployment of electronic devices and cutting-edge electronic systems have transformed the high-tech applications in the modern societies. Among many unresolved concerns, the reliability assessment and lifetime prediction of solder joints in electronic devices have remained a long lasting challenge for researchers, engineers, and developers^1–3^. Among several external factors influencing the reliability of solder joints, thermal cycling is a main one mostly involving every electronic system^4,5^. Many physical models have been proposed so far to tackle the existing challenges on reliability assessment of solder joints^6–9^. A wide range of factors including the fundamental mechanical properties, thermal variations, intermetallic formation, phase transition and defects nucleation have been investigated to predict the probability that the solder interconnection renders an appropriate mechanical and physical behavior in a specific timespan with no failure^10–12^. For instance, great works have been carried out to capture a broad range of temperature and strain rate in the constitutive models^13,14^. These developments undid the existing inconsistencies in the conventional models and rationalized the predictive framework by connecting the parameters such as Young module, ultimate strength and plastic deformation to each other. Considering thermal cyclic rates, novel acceleration-factor equations were also proposed to predict the reliability of solder joints^15^. Mechanics-based acceleration is another approach merging thermal cycling method and mechanical loading to acceptably evaluate the fatigue life of electronic packaging structures^16^. With all these descriptions, since it is implausible to enfold all the contributing factors into a single physical framework, a yawning gap between the model outcomes and experimental tests exist, making it a yet to be solved challenge to achieve a high-fidelity thermal fatigue lifetime prediction. A few statistical techniques are applied, among which the Weibull distribution stands out^17^. In general, Weibull distribution is a particular class of continuous probability density function interpolating between the continuous Rayleigh and exponential distributions with which, the researchers found ways to statistically evaluate the degradation behavior of solder joints under thermomechanical cycling. For instance, Hamasha et al.^18^ indicated that the statistical uncertainty in the operational lifetime of solder joints under a realistic service condition is higher than that estimated in the experimental cycling process. Raj et al.^19^ proposed a statistical methodology, i.e., the Cox proportional hazard regression analysis, to properly evaluate the reliability of solder joints, where they found that the chemical compositions and aging patterns of solder materials are the primary factors affecting the failure rate of the interconnection. Similarly, Ma et al.^20^ declared that the failure rate in the solder joints strongly relies on the package size and the metallurgical aspect of the solder material. Through a typical Weibull analysis, Dalton et al.^21^ reported that the solder chemical composition, temperature change within a thermal cycle, along with a maximum dwell temperature remain the crucial parameters for characteristic lifetimes. Employing Weibull-distributed data and non-linear hybrid models, Berni et al.^22^ claimed that the surface finishes of the printed circuit boards along with the geometry of the joint in common are the dominant influencers on the failure mechanism of the solder interconnection. Other related works typically focused on the reliability assessment of solder joints via conventional statistical methodologies^23–25^. Additionally, particular standards such as the MIL-HDBK-217^26^ and IEEE 1,413.1^27^ present the key statistical factors and methodologies for reliability assessment. Reviewing the existing literature, a number of inconsistencies can be found in the declaration of essential factors in the reliability assessment of solder joints. This observation is primarily supported by the fact that each work emphasized a limited set of parameters for lifetime predictions. Artificial intelligence can be a promising alternative solution to reliability assessments^28,29^. Artificial intelligence models discover a direct relation between the user-defined features of specific components in a system and the external factors. Adequate training datasets create an opportunity to develop predictive models for reliability assessment of mission-critical systems. While a large number of works has been recently devoted to the use of artificial intelligence in power electronics^30–33^, there have been only a few applied to reliability and lifetime prediction of solder joints in electronic systems. In a recent work by Yi and Jones^34^, it was demonstrated that the predicted failure mode via artificial intelligence mechanisms is much more accurate than the conventional statistical methods.

There exist several substantial competing factors contributing to the degradation of the solder joints. Both individual and mutual impacts of such factors, e.g., the chemical compositions, temperature profiles and other physical properties, on the solder joint degradation have been widely accepted in the previous literature^24,35–38^. We propose and validate an artificial intelligence approach for reliability assessment of the solder joints, a novel correlation-driven neural network (CDNN) approach. The proposed CDNN approach holistically captures the mutual interactions of the competing factors, resulting in both high-fidelity estimate of the solder joints’ useful lifetime and an accelerated discovery in predictive models for solder joint reliability evaluation.

The remainder of this paper is structured as follows. Section II introduces the proposed methodology by ceremoniously presenting diverse aspects of the novel algorithm. Numerical results are expressed and extensively discussed in Section III. Finally, conclusions are drawn in Section IV.

## Proposed methodology

**Figure 1:**
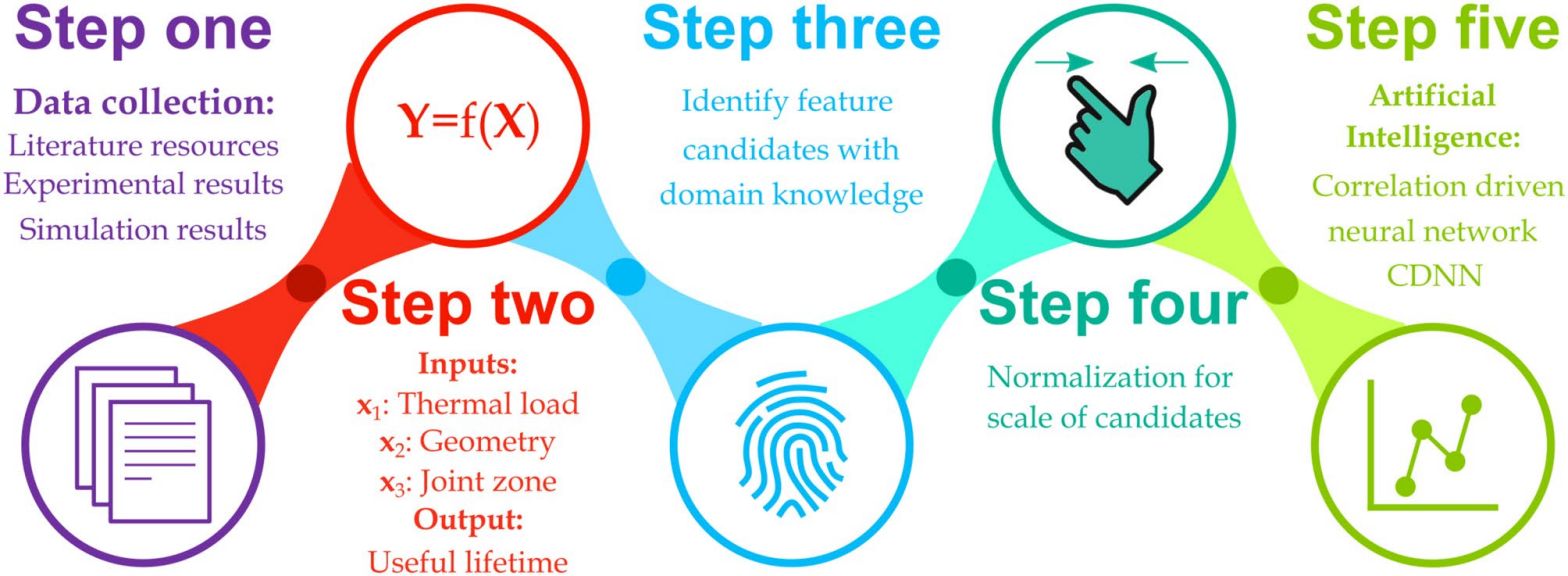
Schematic describing the process of the proposed correlation-driven neural network (CDNN). The process is commenced with data collection from diverse sources and experimental and simulation observations in the literature. Next, the data are categorized into three different sets as the available inputs and useful lifetime as the output in Step two. The potential feature candidates are extracted from the inputs and scaled down to be compatible with our proposed CDNN model. Finally, our proposed network is trained using the processed data.

The cognitive process of an artificial intelligence mechanism falls into five main steps as illustrated in Fig. 1. Following the data collection process and input–output characterization, step three sets forth the feature candidates of materials affecting the useful lifetime of the solder joints in electronic devices. The input data are properly processed and categorized into three diverse sets including composition (joint zone properties), thermal loading specifications, and solder joint geometry. The next step is to use artificial intelligence algorithms to train a predictive model for useful lifetime estimation of solder joints.

### Data collection

Collection of sufficiently large input data plays an important role on the training process. Thanks to the publicity available resources^5,39–45^, we also created a huge number of input data to enrich the data resource needed to train the proposed model. It is worth-mentioning that all the collected data are extracted from the finite element model (FEM) simulation outcomes. The units of all parameters have been unified. The details of FEM procedure, done by authors, are given in Ref.^39,44^. From each resource, we gathered the input datasets including composition (material and physical properties of the joint zone), thermal loading specifications and solder joint geometry. Useful lifetime of each solder joint was recorded and used in the training process (see supplementary data). It is worth–mentioning that most common solder compositions with broad applications were considered for data collection. In recent years, novel solder materials with nanoparticles have been developed^46–50^; however, there exist no sufficient data to consider them in the proposed artificial intelligence approach.

As it was hitherto mentioned, we employed three specific datasets to characterize the input and output factors for training the predictive model. Each of these datasets encompasses several contributory parameters involved in solder joint degradation. Several crucial parameters may impose severe impacts on the lifetime of the solder joints. These parameters include chemical composition (physical and material properties of solder joint), thermal load specifications, geometry features, etc.^51,52^.

One most important factor contributing significantly to the solder joint degradation is the external forces such as thermal loading^2,5,53–55^. Several feature candidates were historically taken into account in order to meticulously characterize the thermal loading. It was extensively reported that the creep and fatigue phenomena are the two most crucial failure mechanisms in the solder joints of electronic devices^2,5^. Accordingly, we consider both of these phenomena in this study. From a physical perspective, continuous variations in thermal loading can naturally cause alternate plastic strains that make the slip band arrests responsible for inner micro stresses causing micro-decohesions. Depending on the type of materials and the loading level, the initiated micro cracks expand either within the crystals or along the boundaries of the grains, up to the coalescence corresponding to the inception of a mesocrack^56^.

Coffin-Manson equation has been employed as the fatigue lifetime model of solder joint^57^. The model is defined as

1 $$N_{f} (\Delta T_{j} ) = A \times \Delta T_{j}^{\alpha }$$

where *A* and α are constant and equipment dependent. *ΔT*_*j*_ describes the temperature change of the devices in the °C junction. *N*_*f*_ is the number of cycles to failure depending on the interpretation of the fault criterion. In general, several developed Coffin-Manson equations with adjunct parameters have been proposed by researchers^58–61^. In the meanwhile, all in common agree that the temperature change in a certain limit, leading to variable ramp rates, plays the most crucial role on the fatigue life of solder joints. Besides, other thermal cycling parameters, i.e., the hot dwell time, not only does have no apparent impacts on the fatigue event, but also may lead to some stress relaxations in the joint zone^62^.

Generally, with the gradual rise in the operating temperature, the time-dependent strength of most materials naturally degrades. The creep failure in common is an event triggered with temperature rise above a one-third of the metal melting point. The described events are exacerbated as the time elapses under the presence of external forces since the high temperatures cause viscous effects on the materials. Time and temperature are also of critical significance in the creep process. Contrary to the fatigue mechanism, creep depreciation is time-dependent and the dwelling time period is tremendously impressive^63^. Of particular note is that the pure creep strain is racked up during the hot dwelling phase, whereas the induced strain during the temperature ramps is mainly uncomprehensive owing to the severe differences in component coefficient of thermal expansion (CTE), which is correlated with the fatigue mechanism^64^. The creep lifetime has been commonly modeled using the Monkman–Grant Equation ^65^:

2 $$\dot{\varepsilon }_{cr} \,\Delta t_{c}^{\beta } = C_{MG}$$

where *C*_*MG*_ and *β* are constant and material-dependent. έ_cr_ is the stable creep strain rate expressed by

3 $$\dot{\varepsilon }_{cr} = C_{1} \left[ {\sinh \left( {C_{2} \sigma } \right)} \right]^{{C_{3} }} \exp \left( {{{ - C_{4} } \mathord{\left/ {\vphantom {{ - C_{4} } T}} \right. \kern-\nulldelimiterspace} T}} \right)$$

where *C*_1_ to *C*_2_ are also constant and material-dependent and are extracted from the creep test. σ is the Von Mises stress in *Pa*. From a viscoplastic point of view, the creep event and plasticity are tightly correlated^13,14^. In many constitutive models, however, the steady-state creep does not behave in the same trend of the rate-dependent plasticity resulting in the accumulated plastic strain not being simply equivalent to the creep strain.

**Figure 2:**
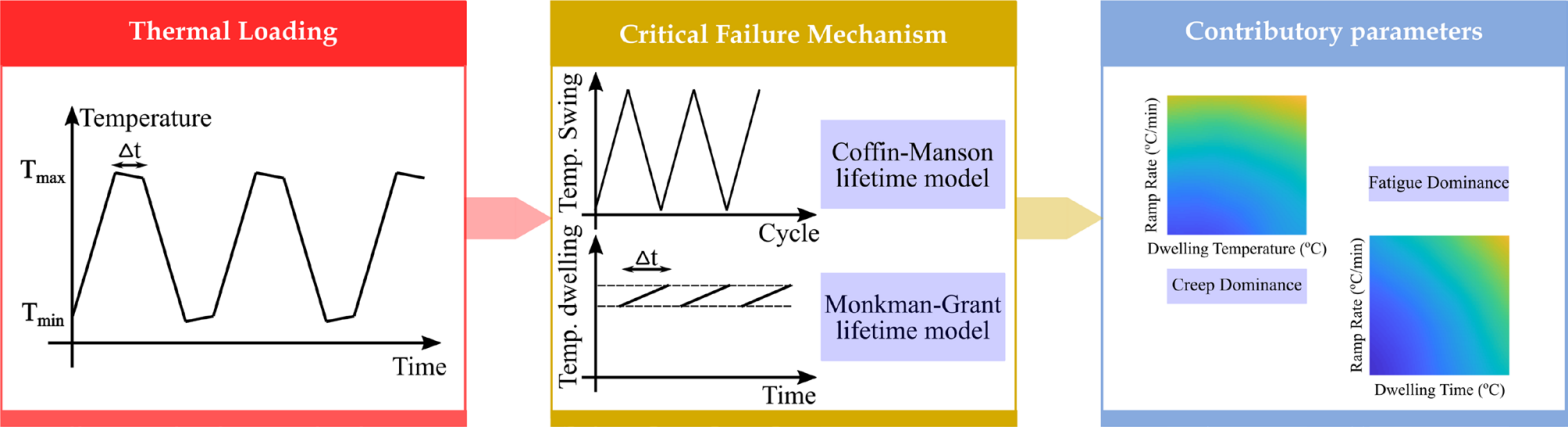
Contributions of the creep and fatigue in solder joint degradation. Thermal cycle decomposes to temperature swing, dwelling temperature, and its corresponded dwelling time. Regarding Eq. (1), temperature swing is the main factor in fatigue failure mechanism, while Eq. (2) and Eq. (3) indicate that dwelling temperature and dwelling time are the main factors in creep failure mechanism.

Previous studies on fatigue assessments were limited to capture only the stress fluctuations and the mean stresses; they mostly neglected to determine the dwelling periods at which the material was efficiently stored at a relatively constant temperature. Figure 2 indicates the thermal stress as a function of time. This is perceived in the thermal stresses; the substance can be subjected to a considerable number of dwelling periods. In such circumstances, consideration of the creep failure process in the lifetime evaluation is significantly needed. Coupled study of creep-fatigue is important due to the range of operating temperatures of the solder. Regarding Eq. (1), Eq. (2), and Eq. (3), one can find that while the contribution of the fatigue failure mechanism in the solder joint degradation heavily depends on the temperature swing *ΔT*_*j*_ (temperature heating up and cooling down rates), the contribution of the creep failure mechanism mainly depends on the dwelling temperature and time. Previous studies have reported that the solder joint geometry plays a key role on its useful lifetime^2,42,66^. Principally, the bulkier the solder material, the more elastic and inelastic strains are induced in the solder material leading to accelerated degradation. We concentrated fiercely on the plate solder joint to accurately classify the training dataset.

Hither, this comprehensive analysis considered two specific sets of data namely the solder joint and the upper/basal plates. Our data collection includes multiple standard and applicable compositions of the solder joint, such as SAC305, SAC387, SAC405, etc.^67,68^. We properly classify the alloy composition as the input into our predictive model since our aim in utilizing these models would be to efficiently determine promising alloy compositions. Owing to their widespread deployment in electronic devices, two separate cases, namely Si/Cu and Si/Al, were adequately taken into account in the upper/basal plates^12,69–71^.

### Feature candidates

Features candidate (**X**) are regarded as the inputs to the CDNN model (f) in order to precisely predict the desired output (**Y**), i.e., **Y** = f(**X**). Therefore, an adequate set of **X** has to be defined for a given target output **Y** to ensure that a well performing CDNN model is produced. The contributory parameters for lifetime estimation of the solder joints include the chemical composition, physical and material properties of solder joint, thermal load specifications, geometry features, etc.^51,52^.

Table 1 lists the contributory factors considered in this study. Well-known Sn–Ag–Cu solder may comprise the adverse compositions, where each distinctive composition may naturally affect the predictive model for useful lifetime estimation of the solder joints. Each of the Sn, Ag and Cu individual percentages as well as their overall combination are important in reliability assessment of the solder joints. Thermal load and geometry specifications may severely influence the solder joint reliability by intensifying either creep or fatigue failure mechanisms. Therefore, such potential parameters were involved in our study.

**Table 1:** The physical properties, thermal load specifications and geometry features.

Thermal load specifications	Joint zone specifications	Geometry
Hot dwelling temp. (°C)	SJ/U/B Melting Temp. (°C)	Thickness (um)
Cold dwelling temp. (°C)	SJ/U/B Poisson ratio	Length (mm)
Hot dwelling time (min)	SJ/U/B Young Module (GPa)	Width (mm)
Cold dwelling time (min)	SJ/U/B CTE (10^–6^/°C)	
Heating rate (°C /min)	SJ/U/B Density (g/cm^3^)	
Cooling rate (°C /min)		
SJ: Solder Joint	U/B: Upper/Basal	Temp: Temperature

Although the possible range of the considered feature candidates has expanded, it might be inappropriate to directly involve them in neural network or any other machine learning algorithms. In response, a data preprocessing, namely feature scaling, is opted for normalizing the feature candidates. The min–max rescaling formula is employed to rescale the feature candidates into a predefined range of [a, b]:

4 $$x_{new} = a + \frac{(b - a)(x - \min (x))}{{\max (x) - \min (x)}}$$

where *x* and *x*_*new*_ are the original and the scaled feature candidate, respectively. *min(x)* and *max(x)* are the minimum and maximum value of the feature candidate over the training dataset, respectively. It has been reported that the smaller the scaling range is, the higher the achieved accuracy will be. Accordingly, we considered *a* = 0.2 and *b* = 0.8^72,73^.

### Correlation-driven neural network model (CDNN)

**Figure 3:**
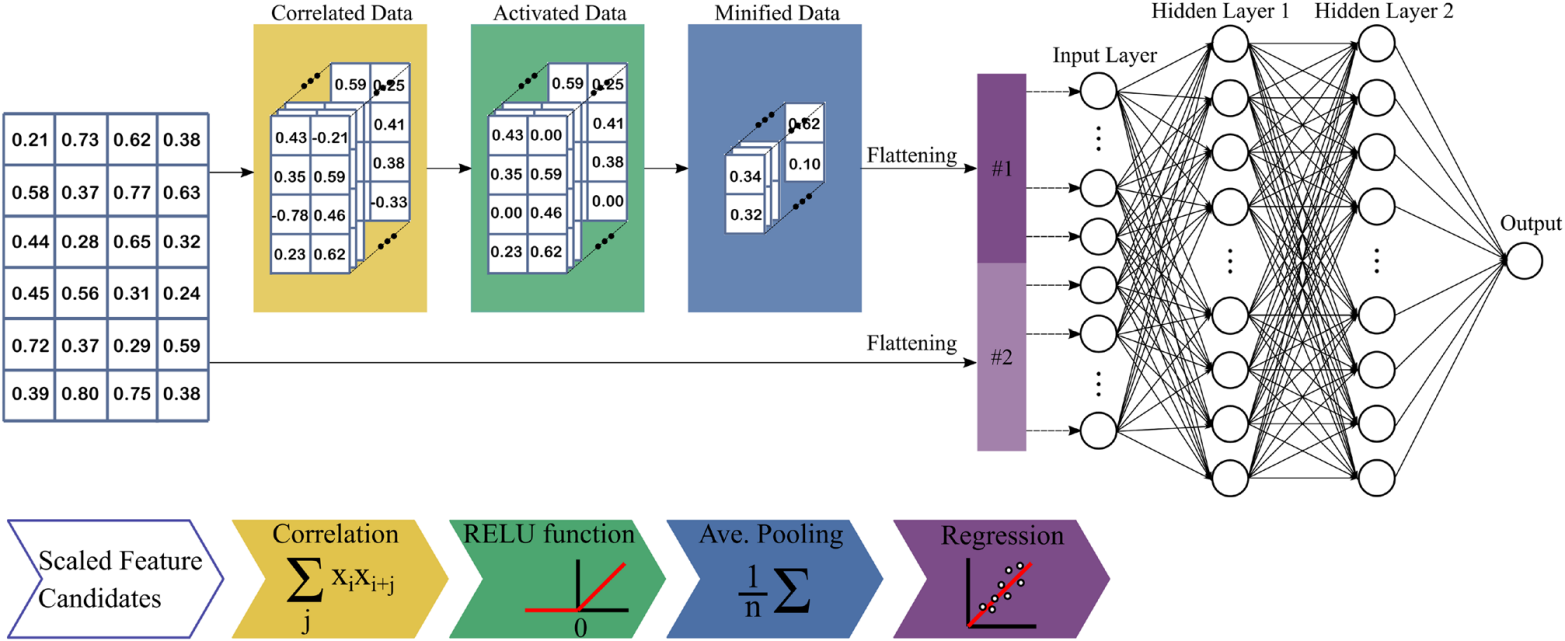
Pictorial description of the proposed CDNN structure. Each dataset was correlated with 80 correlation 3*3 matrices, the outputs were nonlinearly activated using Rectified Linear Unit (ReLU) functions and then minified with average pooling. A total of 160 flattened features in addition to the initial 24 scaled feature candidates were generated. 184 generated features were utilized for training the 2-level fully connected neural network. The training process tries to obtain the weights of the fully connected neural network and also find the 80 correlation 3*3 matrices.

There are many Machine Learning (ML) algorithms, such as the linear ML algorithms, nonlinear ML algorithms and ensemble ML algorithms that have been commonly used in the previous literature^72,74^. It has been extensively reported that the creep-fatigue failure mechanisms play significant roles in the solder joint degradation^2,5,53^. Both these phenomena are physically triggered by multiple aforementioned contributory factors (see Table 1). The main deriving forces in activating these factors are the elastic and inelastic strains occurring in the power device body, especially in the solder joint. The induced elastic and inelastic strains are originated from the thermomechanical stresses which are fundamentally actuated by the CTE differences in the joint zones. It is reported that there exist dependencies and correlations among contributory parameters in creep-fatigue occurrences^5,8^. For instance, dwelling temperature and time individually and mutually affect the induced inelastic strain in the solder joint^53–55^. Accordingly, the individual and mutual interactions of these two feature candidates have to be captured in a holistic ML mechanism to accurately predict the useful lifetime of the solder joint. In this study, we propose a new artificial intelligence algorithm called correlation driven neural network (CDNN), which captures the correlations among the feature candidates. Thanks to CDNN, a more accurate predictive model (f) is mapped between the inputs (or features) and the output (objectives) for reliability assessment of solder joint in electronic devices. Pictorial description of the proposed CDNN architecture is shown in Fig. 3. Globally, it contains two unique paths and follows by a deep neural network, usually known as fully connected layers (FCL). A path belongs to the correlated data in which the input feature candidates correlate with each other via the trained correlation matrices (the elements would be derived in the training process). CDNN may have stack of multiple correlating, activating and pooling layers. Predictably, the more layers are employed, the less error occurs at the expense of a longer computational time. The second path directly transfers the feature candidates to the deep neural network. Thus, both individual and mutual effects of the feature candidates are properly considered, leading to a more accurate estimation outcome. Considering the correlation of feature candidates will result in a more precise and quicker CDNN in comparison with the conventional neural network. We will describe the methods in the following sections.

Cross-correlation of the feature candidates is the core component of the proposed CDNN, which is achieved through several correlating matrices. The first layer receives the input scaled feature candidates. Then, the first layer performs cross-correlation of the scaled feature candidates with several correlation matrices ultimately resulting in multiple correlated datasets. Regarding the number of correlation matrices, each correlated dataset determines a specific feature. The correlating layer employed in this process can be mathematically expressed as follows

5 $${\varvec{y}}[i,j] = \sum\limits_{a = 0}^{c - 1} {\sum\limits_{b = 0}^{r - 1} {{\varvec{w}}[a,b]{\varvec{x}}[i + a,j + b]} }$$

where ***w*** and ***x*** are the correlating matrix and the input scaled feature candidate matrix, respectively. ***y ***[*i*,*j*] presents a discrete output of the correlating layer. *c* and *r* are the column and the row of the correlating matrix (***w***). The output dimensions are calculated as *P*_***y***_ = *P*_***x***_* –P*_***w***_ + 1 where *p* indicates the row or the column of a matrix (for having the maximum overlapping among the feature candidates, a stride of one is adopted in the correlating layer). It is notable that zero-padding may also be used for unifying the data size.

In the correlating layer, the output ***y*** from Eq. (5) is progressed to a nonlinear activation function to strengthen the predictive model. Here, a Rectified Linear Unit (ReLU) activation function is used as follows^75,76^:

6 $$\text{Re} LU(x) = \left\{ {\begin{array}{*{20}c} 0 & {if\,x < 0} \\ x & {if\,x \ge 0} \\ \end{array} } \right.$$

Since the correlating layer only involves a linear mapping process, the activation function is to capture the input–output nonlinear complex functional mappings. This activation function is applied to all the correlated datasets. Consequently, many resulting correlated datasets are projected to efficiently capture a broad range of salient correlated features.

A down sampling operation called pooling layer is also embedded in the proposed CDNN to minify the correlated data over each dataset, while preserving the most important information in the data. Minifying operation results in fewer data processed by the correlating layer. The average pooling is one of the most commonly used pooling procedures, which basically casts to matrix coarsening. This can be mathematically expressed as follows

7 $$Pool(x,y) = \sum\limits_{j = 0}^{q - 1} {\sum\limits_{i = 0}^{q - 1} {\frac{I(q \times x + i,q \times y + j)}{{q^{2} }}} }$$

where *q* denotes the coarsening length scale, and (*x*,*y*) determines the output of the correlated data following the average pooling applied. The pooling layer is applied for each correlated dataset. The pooling application significantly relaxes the computational complexity of the predictive model training.

The minified data (data group #1) as well as the scaled feature candidates (data group #2) are flattened to a one dimensional vector as shown in Fig. 3. The joint-data are then inserted into the conventional artificial neural network (CANN). CANN is a multilayer network, with one-input one-output structure and one or more hidden layers. Each of these layers processes the information in its previous layers via multiple neurons. A layer is formulated as follows^77^

8 $$\gamma _{i}^{\ell } = f\left( {\sum\limits_{{j = 1}}^{{N_{{\ell - 1}} }} {\omega _{{ij}}^{l} \,\,\gamma _{i}^{{\ell - 1}} + b_{i} } } \right)\quad i = 1,\,...\,,\,N_{\ell }$$

where *γ*^*l*^_*i*_ is the output of the *ith* neuron in the *lth* layer. *N*_*l*_ and *N*_*l-1*_ are the number of the *lth* and (*l-1)th* layers, respectively. *ω*^*l*^_*ij*_ is the weight factors correlating the inputs to a certain output. As in the correlating layer, *f* is an activation function in the fully connected layer. Sigmoid, ReLU and Softmax are the most common activation functions used in nonlinear regression and classification problems. *b*_*i*_ is its bias value and can take any arbitrary value. CANN is a universal function estimator and is potentially able to vary the weights and bias terms in its structure to predict any input/output data relationships with an arbitrary precision. Throughout the training cycle, these parameters are modified so as to implementing the typical back-propagation algorithm.

The performance of the identification models are carefully assessed through the lenses of the model precision, i.e., the proportion of the samples properly identified. The performance of the regression models is evaluated using the correlation factor *r* as follows^72^

9 $$r = \sqrt {{{\sum\nolimits_{i = 1}^{n} {\left( {\hat{y}_{i} - \overline{y}} \right)^{2} } } \mathord{\left/ {\vphantom {{\sum\nolimits_{i = 1}^{n} {\left( {\hat{y}_{i} - \overline{y}} \right)^{2} } } {\sum\nolimits_{i = 1}^{n} {\left( {y_{i} - \overline{y}} \right)^{2} } }}} \right. \kern-\nulldelimiterspace} {\sum\nolimits_{i = 1}^{n} {\left( {y_{i} - \overline{y}} \right)^{2} } }}}$$

and the root mean square error (RMSE)

10 $$RMSE = \sqrt {\sum\limits_{i = 1}^{n} {\frac{1}{n}\left( {\hat{y}_{i} - y_{i} } \right)^{2} } }$$

where ${\widehat{y}}_{i}$, ${y}_{i}$ and $\stackrel{-}{y}$ are predicted, actual, and the mean value of the actual output, respectively. *r* values lies in [0 1] with 1 representing a perfect fitting.

## Results and discussions

**Figure 4:**
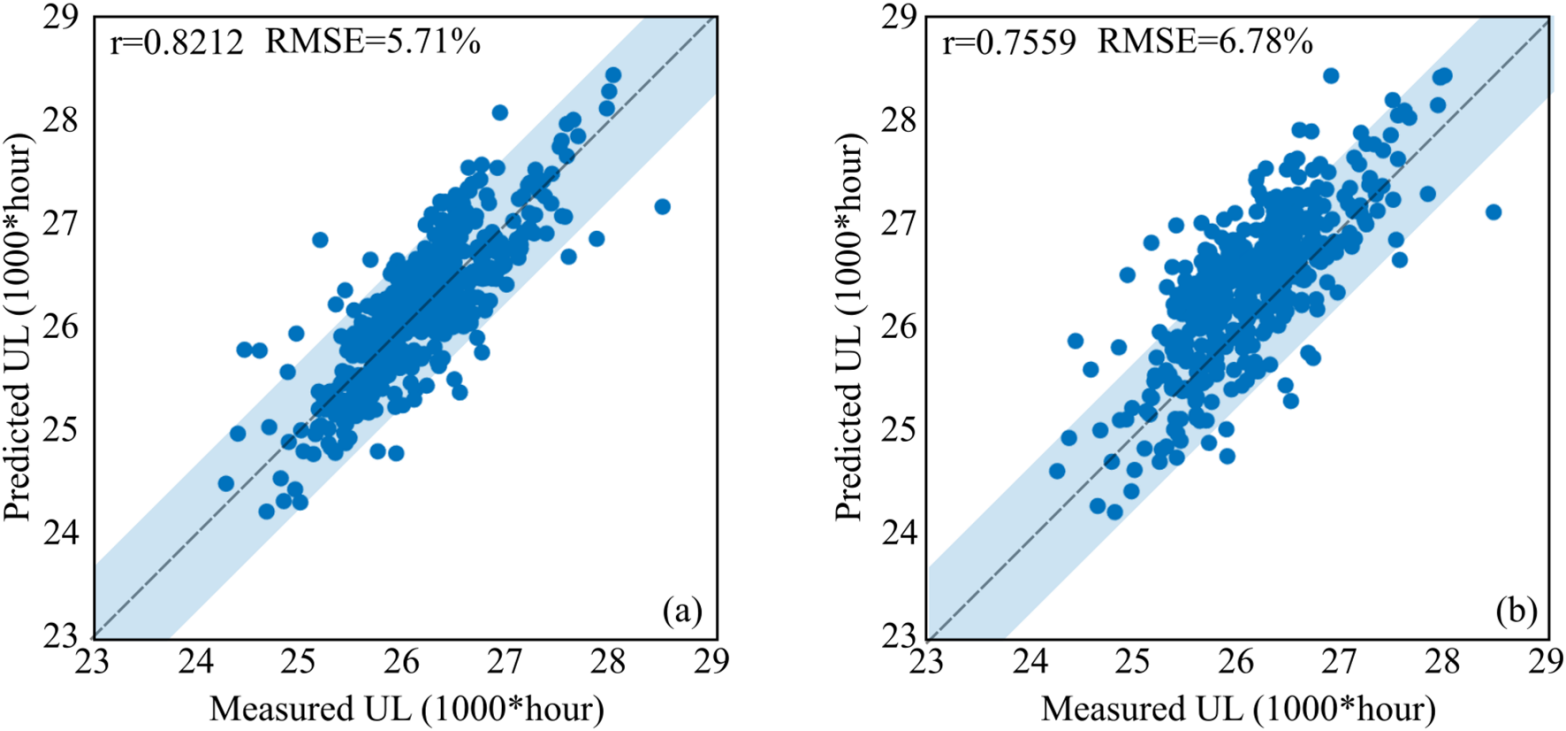
Neural network predicted values versus the measured values. (**a**) Proposed correlation-driven neural network (CDNN), (**b**) Conventional artificial neural network (CANN).

The first objective in our predictive framework is to establish an estimation of the CDNN model precision. The plots of the CDNN- and CANN-predicted useful lifetime (UL) of the solder joints against the measured values are given in Fig. 4. It is found that our proposed CDNN model renders a more acceptable predictive performance in comparison with the CANN approach. The CDNN model achieved the RMSE value of 5.71%, while that of the CANN model is reported 6.78%. Moreover, the performance of the regression model in CDNN is meaningfully higher than that of the CANN, indicating that our model includes a set of learning predictive regulation accurately carrying over from one thermal cycling system to another. This observation is principally originated from the joint consideration of the mutual and individual interactions of the feature candidates in the proposed neural network. As mentioned, several correlation matrices were employed in the proposed CDNN model to capture the mutual impacts of the feature candidates on the useful lifetime estimates of the solder joints. Nevertheless, there are some outliers far from the critical zone of prediction in both models. Evaluating the numerous feature candidates in our model, it is revealed that the type of the base materials joined by the solder layer leads to the largest error. The CDNN model extremely overestimates the reliability of the system when the base materials are aluminum and silicon chip. This large error is due to the few number of Si/solder/Al junction entries in the dataset. Interestingly, the extreme underestimation of the CDNN model also belongs to the Si/solder/Al junction. When the solder joint is much thicker (60 μm), the model overestimate the UL of the system, while an underestimation happens when the solder layer at Si/solder/Al interconnection is too thin (20 μm). Except the mentioned joint with a low number of reported data, some few points are yet found outside the highlighted region, which may be due to the inaccuracy in the simulation outcomes.

**Figure 5:**
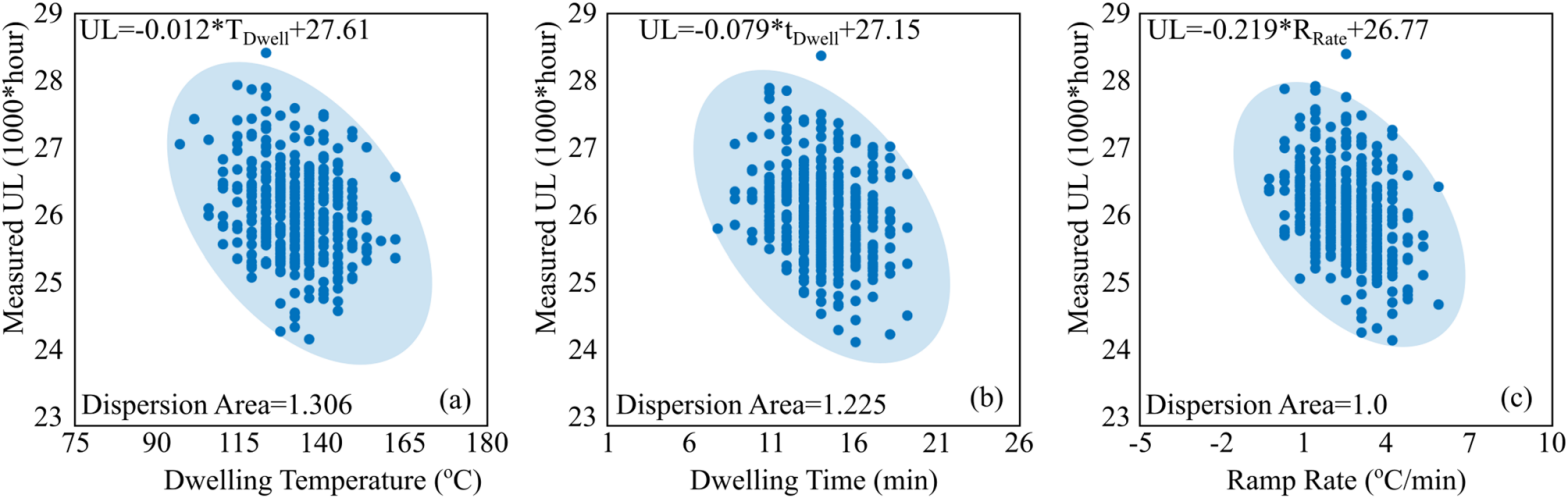
Relation between the measured UL and three main feature candidates, which are significantly affecting the reliability of a solder joint under a thermal cycling process. (**a**) Useful lifetime versus dwelling temperature, (**b**) useful lifetime versus dwelling time and (**c**) useful lifetime versus ramp rate. Dispersion areas are normalized to that of the ramp rate-UL.

Figure 5 illustrates the relation between the measured UL and three main features significantly affecting the reliability of a solder joint under a thermal cycling process. The boundary conditions are based on the collected input data in which dwelling temperatures, dwelling times and ramp rates are ranged within [90 165] °C, [6 18]min and [0.5 6] °C/min, respectively. As observed, the ramp rate considerably influences the reliability of the solder joints in the electronic devices, while the dwelling time and the temperature parameters have more moderate impacts on the UL. Moreover, the trend of the points’ distribution in Fig. 5 indicates that the configuration of the ramping-UL plot tends to constringe in a smaller ellipse, highlighting that the ramping parameter is more independent of the dwelling time and temperature. On the other side, the trends in Fig. 5b,c reveal that the temperature and dwelling time in a thermal cycle profile strongly rely on each other and on the materials properties of the components in the electronic device. In general, a thermal cycling process leads to the creep-fatigue failure in the solder joint. Under this condition, the hot dwelling parts of a thermal cycle including the dwelling time and its corresponding temperature are indicators of the creep event and provides driving forces for visco-plastic strain damage in the solder interconnection, while the ramping part mainly derives the fatigue failure mechanism^8,78,79^. However, it of note that these two events, i.e., the creep and fatigue, in a thermomechanical process are interwoven and it is difficult to easily distinguish them. Moreover, due to the separated portion of the creep event in a thermal cycle profile, i.e., the dwelling time and temperature (see Fig. 5a,b), one should notice that the sharper effects of the ramp rate on the UL do not necessarily indicate the dominant role of fatigue in a failure occurrence.

**Figure 6:**
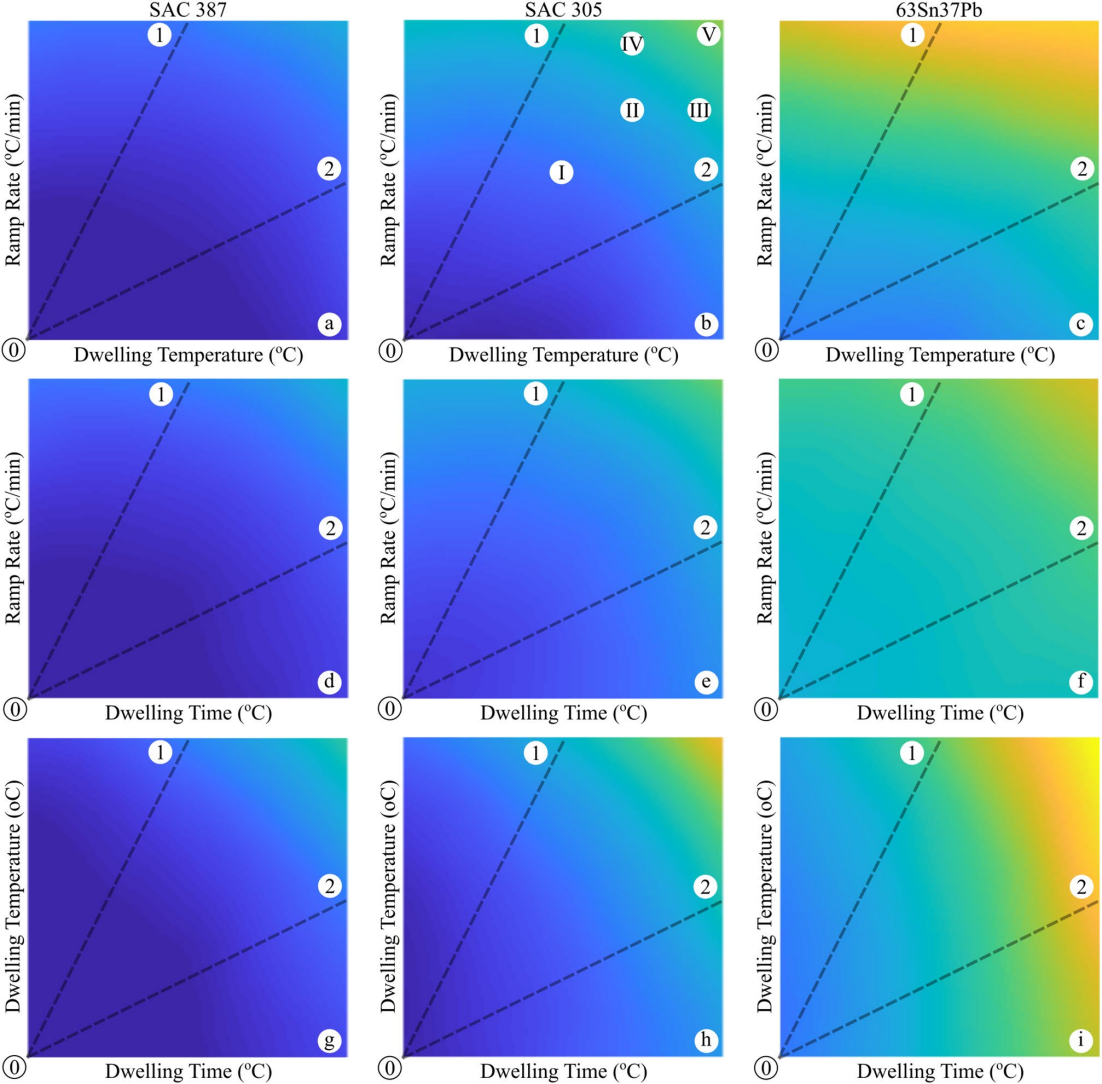
UL prediction in three diverse solders (SAC387, SAC305 and 63Sn37Pb). Blue to yellow colors show the maximum to the minimum predicted UL as functions of ramp rate, dwelling temperature, and dwelling time. Ramp rate, dwelling temperature, and dwelling time are all varying from their minimum to their maximums values, which were scaled down to the range of [0.2 0.8]. Lines 0–1 and 0–2 are highlighting the dominance of variation of y-axis and x-axis, respectively. Points I, II and V are denoting UL predictions in which the ramp rate and dwelling temperature both share identical contributions. Points III and IV are denoting UL predictions in which the dwelling temperature and ramp rate share more significant contributions, respectively.

**Figure 7:**
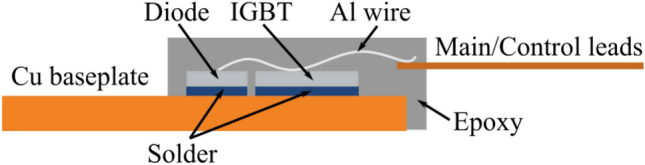
Wire bonding power semiconductor structure.

Figure 6 presents the role of the ramp rate, dwelling temperature, and time on the predicted UL of three known solder joints in a unique electronic device with the same components and geometries (see the physical structure of a common discrete power semiconductor in Fig. 7). As observed, the solders, i.e., the SAC387, SAC305 and SnPb, with distinct materials properties well demonstrate the effects of the thermal cycling parameters on the reliability of an electronic device. At the first glance, one can readily see that with the increase in the values of the thermal cycling parameters, the fatigue lifetime declines in all the solder types. However, the SnPb solder suffers the most from the intensified thermal cycle. On the other side, the SAC387 and SAC305 solders show closer behaviors under a thermal cycling process. Quantitatively, we select the ramp rate of 3.5 °C/min, hot dwell time and temperature of 12 min and 125 °C, which are approximately located at the center region of the contour plots in Fig. 6. With these coordinated points, the UL of 27,788, 27,213 and 26,153 h were measured for SAC387, SAC305 and SnPb, respectively, which implies high reliability performance of SAC387 in the thermal cycling process. Accordingly, the portion of the fatigue and creep events on the failure evolution of the solder joints is still obscure. In order to further clarify, we track the trend of the UL according to the dashed lines drawn in the plots. The UL values along the dashed lines will display the effects of the thermal cycling parameters. Table 2 lists the UL variations of a solder joint as the function of ramp rate, dwelling time, and temperature as the input thermal loading in different solder layers, i.e. SAC 387, SAC305 and 63Sn37Pb. Considering the temperature–time plots and based on the recorded values (see Table 2), the declining trend of UL from the left corner side (common beginning points of the dashed lines) is found approximately similar for two dashed lines in all the three solder types except SnPb in which the dwelling temperature looks a bit more dominant owing to its lower Young module. This indicates that the influence of the hot dwelling temperature and time under a certain ramp rate is somehow alike. On the other side, the type of the solder plays an important role when the ramp rate compares to the dwelling time and temperature. For the SAC305, the rate of the declining UL across the dashed line 0–2 is approximately the same as that for the dashed line 0–1 (Fig. 6b,e, Table 2). This means that the ramp rate has an equal impact on the failure evolution of the solder joints made by SAC305. This event was clearly seen in the SAC387 so that a balance appeared between the dwelling time, temperature and the ramping values (Fig. 6a,d). Instead, the SnPb solder shows a severe behavior, so the declining trend across the dashed line 0–1 is significantly higher than that for dashed line 0–2 (Fig. 6c). Hence, one can conclude that the dwelling parameters are more critical in assemblies made by SnPb solder. The philosophy of these contradictory events relate to the inherent properties of the solder alloys and other components in an assembly. The solders SAC305 and SAC387 with CTEs of 21.6 × 10^–6^ m/°C and 20 × 10^-6^ m/°C and melting temperatures of 220 °C and 217 °C demonstrate a higher resistance against the creep event in the joint zone. On the other hand, the PbSn solder with low a melting point of 183 °C is very sensitive to the creep event; that is with the increase in the hot dwelling time and temperature, the driving force for the elemental inter-diffusion, void formation and growth, and the glide of dislocations is provided and the creep failure prevails in the damage evolution. In summary, with the same parameters, the SnPb solder with lower melting point and young module is weaker than the SAC solders; however, the role of the creep damage in this solder is more intensive than the fatigue event. On the other side, the SAC solders with higher CTE values are found more susceptible to the fatigue damage during a thermal cycling process in comparison with the SnPb solder.

**Table 2:** Useful lifetime of the solder joint under different parameters uncertainty such as in the ramp rate, dwelling time and temperature in three different solder joints, namely SAC387 (Fig. 6a,d,g), SAC305 (Fig. 6b,e,h) and 63SnPb37 (Fig. 6c,f,i).

Begins from	a		b		c		d		e		f		g		h		i	
	⓪		⓪		⓪		⓪		⓪		⓪		⓪		⓪		⓪	
Parameters increasing from their minimum to their maximum scaled with 0.2 to 0.8	0.7893	0.7893	0.7415	0.7415	0.6069	0.6069	0.7766	0.7766	0.7122	0.7122	0.5161	0.5161	0.8000	0.8000	0.7383	0.7383	0.6135	0.6135
	0.7706	0.7737	0.7147	0.7265	0.5640	0.5826	0.7598	0.7582	0.6990	0.7018	0.5062	0.5063	0.7858	0.7777	0.7268	0.7129	0.5968	0.5721
	0.7613	0.7641	0.6976	0.7183	0.5415	0.5725	0.7545	0.7523	0.6872	0.6931	0.5007	0.5014	0.7760	0.7683	0.7169	0.6985	0.5812	0.5469
	0.7469	0.7556	0.6767	0.7016	0.5174	0.5598	0.7441	0.7499	0.6700	0.6812	0.4944	0.4950	0.7629	0.7574	0.7027	0.6825	0.5658	0.5176
	0.7301	0.7450	0.6497	0.6868	0.4895	0.5435	0.7299	0.7431	0.6495	0.6624	0.4883	0.4860	0.7454	0.7433	0.6868	0.6624	0.5437	0.4871
	0.7134	0.7342	0.6222	0.6669	0.4532	0.5234	0.7149	0.7293	0.6289	0.6399	0.4789	0.4766	0.7287	0.7245	0.6664	0.6358	0.5229	0.4553
	0.6928	0.7181	0.5913	0.6393	0.4141	0.5009	0.6976	0.7166	0.6060	0.6189	0.4668	0.4653	0.7130	0.7010	0.6330	0.6027	0.5043	0.4182
	0.6685	0.7000	0.5581	0.6068	0.3755	0.4756	0.6745	0.6992	0.5794	0.5951	0.4499	0.4559	0.6866	0.6748	0.5949	0.5649	0.4775	0.3764
	0.6417	0.6743	0.5181	0.5689	0.3284	0.4488	0.6392	0.6740	0.5454	0.5635	0.4263	0.4384	0.6485	0.6463	0.5517	0.5225	0.4415	0.3312
	0.6101	0.6424	0.4699	0.5293	0.2834	0.4191	0.6000	0.6425	0.5060	0.5290	0.4021	0.4183	0.6010	0.6108	0.5066	0.4706	0.4037	0.2838
Ends at	①	②	①	②	①	②	①	②	①	②	①	②	①	②	①	②	①	②

ULs are scaled between 0.2 and 0.8.

**Figure 8:**
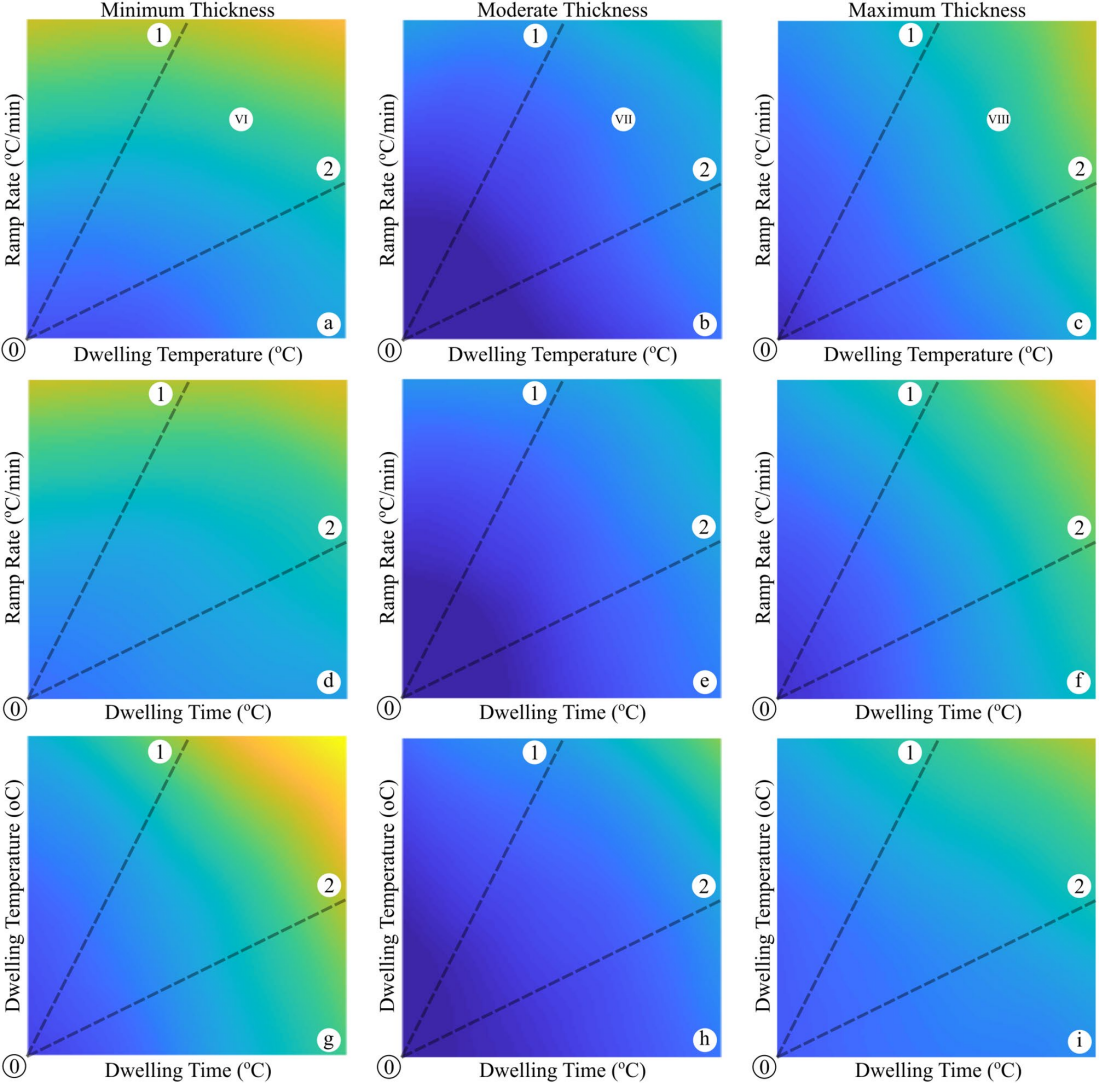
UL prediction in three diverse solder layer thicknesses. Blue to yellow colors show the maximum to the minimum predicted UL as functions of ramp rate, dwelling temperature, and dwelling time. Ramp rate, dwelling temperature, and dwelling time are all varying from their minimums to their maximums, which were scaled down to the range of [0.2 0.8]. Lines 0–1 and 0–2 are highlighting the dominance of variation of y-axis and x-axis, respectively.

With a geometrical approach, the solder layer thickness is one of the most important variables affecting the reliability of the electronic devices^2,5^. Therefore, we analyzed an electronic assembly similar to that considered in Fig. 6. Figure 8 represents the plots of the dwelling time, temperature and the ramp rate correlated with SAC405 solder with different thicknesses. All the assembly parameters are constant while the solder thickness solely changes. The UL of the SAC405 solder with the mean normalized thermal cycling parameters are found 26,456, 27,393 and 26,688 h for 20, 40 and 60 µm thickness, respectively. This observation indicates that an optimal selection of the solder thickness may improve the reliability of the electronic device. The trend of the UL values with the dashed-line procedure, described in Fig. 6, was also evaluated. Table 3 lists the UL variations of the solder joint as the function of ramp rate, dwelling time, and temperature as the input thermal loading in different solder layer thicknesses, i.e. 20, 40 and 60 µm. According to the results (see Table 3), the thin solder (20 µm) extremely suffers from the dwelling time and temperature, highlighting that the creep event would be dominant on the failure evolution. With the increase in the thickness to 40 µm, both fatigue and creep processes equally get involved in the damage evolution of the solder joint. On the other side, the thicker the solder layer is, the higher resistance to the creep failure is observed, while more affected by the fatigue process. Hence, it is found that the optimal solder layer (40 µm) provides a reliable junction, which is moderately touched with the fatigue and creep processes, while the thicker and thinner layers are heterogeneously influenced by the thermal cycling parameters. This observation is primarily due the fact that the change in the thickness/volume of the solder alters the values and the distribution of the accumulated energy in the junction^2,12^. At the thin solder, the thermal cycling leads to generation of some creep deformation in the junction layer. This viscoplastic strain, which is created at the hot dwelling temperature, is stored as a strain energy in the volume of the solder layer. This strain energy induces both the creep and fatigue mechanisms in the solder joint. With the same parameters, it is concluded that the thin solder (20 µm) experienced the maximum stored strain energy in the junction layer, which may lead to a lower UL and a catastrophic failure. With the thick solder (60 µm), however, the accumulated creep energy significantly decreases in the volume of the junction layer, resulting in a higher UL compared to the 20-µm-thick solder. The high thickness of the solder highlights the role of the CTE mismatch in the interconnection and the effect of the fatigue event in comparison with the creep mechanism. Eventually, the junction is highly reliable when a balance exists between the stored strain energy per volume and the fatigue mechanism, as manifested in a 40-µm-thick solder case study.

**Table 3:** Useful lifetime of the solder joint under different parameters uncertainty such as in the ramp rate, dwelling time and temperature in three different solder joint thicknesses, namely 20um (Fig. 6a,d,g), 40um (Fig. 6b,e,h) and 60 um (Fig. 6c,f,i).

Begins from	a		b		c		d		e		f		g		h		i	
	⓪		⓪		⓪		⓪		⓪		⓪		⓪		⓪		⓪	
Parameters increasing from their minimum to their maximum scaled with 0.2 to 0.8	0.6717	0.6717	0.8000	0.8000	0.7303	0.7303	0.6268	0.6268	0.7773	0.7773	0.7159	0.7159	0.6926	0.6926	0.7558	0.7558	0.6564	0.6564
	0.6273	0.6517	0.7700	0.7723	0.6854	0.6781	0.5956	0.6030	0.7678	0.7602	0.6919	0.6836	0.6596	0.6546	0.7463	0.7366	0.6424	0.6340
	0.6034	0.6368	0.7533	0.7511	0.6627	0.6521	0.5768	0.5871	0.7511	0.7437	0.6718	0.6576	0.6393	0.6279	0.7344	0.7224	0.6309	0.6228
	0.5692	0.6152	0.7278	0.7226	0.6454	0.6302	0.5544	0.5690	0.7232	0.7189	0.6509	0.6273	0.6166	0.5912	0.7135	0.7051	0.6208	0.6104
	0.5323	0.5906	0.6978	0.6908	0.6217	0.5983	0.5318	0.5507	0.6976	0.6881	0.6222	0.5945	0.5907	0.5520	0.6904	0.6873	0.5977	0.5937
	0.4994	0.5655	0.6745	0.6619	0.5949	0.5613	0.5033	0.5349	0.6761	0.6572	0.5872	0.5605	0.5600	0.5141	0.6700	0.6700	0.5666	0.5776
	0.4645	0.5361	0.6512	0.6302	0.5643	0.5242	0.4685	0.5175	0.6534	0.6327	0.5506	0.5220	0.5224	0.4791	0.6455	0.6491	0.5331	0.5577
	0.4290	0.5105	0.6218	0.6001	0.5325	0.4848	0.4314	0.4977	0.6206	0.6081	0.5084	0.4819	0.4736	0.4395	0.6191	0.6239	0.4991	0.5326
	0.3882	0.4862	0.5866	0.5716	0.4995	0.4421	0.3952	0.4780	0.5887	0.5750	0.4727	0.4425	0.4159	0.3949	0.5894	0.5878	0.4680	0.4994
	0.3468	0.4555	0.5424	0.5419	0.4710	0.4043	0.3551	0.4551	0.5505	0.5422	0.4375	0.4046	0.3570	0.3478	0.5506	0.5426	0.4371	0.4710
Ends at	①	②	①	②	①	②	①	②	①	②	①	②	①	②	①	②	①	②

ULs are scaled between 0.2 and 0.8.

In order to validate the predicted values extracted from the proposed CDNN approach, eight different case studies were considered and simulated through a finite element modeling approach (see Table 4). It should be noted that the studied cases were not included in the input data and were independently applied solely for verifications. The initial conditions were kept identical in both numerical and simulation assessments. The cases, namely I to V, are depicted in Fig. 6b. The results, listed in Table 4, indicate the high accuracy of the proposed CDNN model over scenarios with diverse creep-fatigue damage dominances. However, one can see that at low and intensified dwell temperature and ramp rates, i.e., cases I and V, the prediction performance slightly decreases. Considering cases VI to VIII marked in Fig. 8, it is also concluded that the predictive model have an acceptable accuracy with the change in solder thickness. Howbeit, the prediction deviates at the thicker joints (case VIII). All the mentioned deviations, even in the lowest value, are due to the fact that the input data at the selected domains were not enough to strengthen the predictive model. With all these descriptions, it is clear that the CDNN model accurately works and well establishes a framework for accelerated reliability assessment. Regarding the outcomes of this study, it is possible to unhand the time consuming FEM simulations and experimental works since our proposed CDNN model achieves the goal in just a few seconds. Moreover, our predictive model provides a perspective for researchers to design new electronic systems with reliable electric junctions being able to have excellent performance in diverse environments with various thermomechanical conditions.

**Table 4:** Performance Comparison of the proposed CDNN and the FEM simulation results in different cases.

Case	CDNN results (hours)	Simulation Results (hours)	Error (%)
I	27,213	26,064	4.2
II	26,470	25,988	1.8
III	26,072	25,208	3.3
IV	25,893	24,987	3.5
V	25,505	23,963	6.0
VI	24,904	25,875	3.9
VII	26,875	26,041	3.1
VIII	25,748	27,653	7.4

## Conclusion

In this paper, a correlation driven neural network (CDNN) mechanism was developed based on the conventional artificial neural network in order to accurately capture the mutual impacts of the main features during the training process. A significant number of data was collected and inserted to the novel CDNN framework. With the mutual impacts of diverse physical parameters mathematically incorporated in the proposed approach, the prediction model demonstrated a much better performance. The root mean square error were found 5.71% and 6.78% for the CDNN and CANN approaches, respectively. As a case study, the effects of solder materials on the failure evolution of the solder joints were discussed. The exact shares of both fatigue and creep events on the damage evolution of the solder joints were extracted and evaluated via the proposed model. In addition, the effects of the solder layer thickness were also investigated and an optimized solder layer thickness was estimated in which the creep and fatigue damages were in balance. A complementary set of simulations also validated the accuracy of the proposed CDNN approach in different creep-fatigue damage dominances.

## Supplementary information

Supplementary information 1

Supplementary information 2
