# Supplementary material for: Correlation-driven machine learning for accelerated reliability assessment of solder joints in electronics
Source: Sci Rep. 2020 Sep 9;10:14821. doi: 10.1038/s41598-020-71926-7 (PMC7481227; doi:10.1038/s41598-020-71926-7)
Supplement: Supplementary file 2 — Supplementary information 2 [file 41598_2020_71926_MOESM2_ESM.docx]

**Correlation-Driven Machine Learning for Accelerated Reliability Assessment of Solder Joints in Electronics**

**Vahid Samavatian^1^, Mahmud Fotuhi-Firuzabad^1^, Majid Samavatian^2^, Payman Dehghanian^3^ and Frede Blaabjerg^4,*^**

^1^ Department of Electrical Engineering, Sharif University of Technology, Tehran 68260, Iran

^2^ Department of Advanced Materials and Renewable Energy, Iranian Research Organization for Science and Technology (IROST), Tehran 33535111, Iran

^3^ Department of Electrical and Computer Engineering, The George Washington University, Washington DC 20052, USA

^4^ Department of Electrical Engineering, Aalborg University, Aalborg 9100, Denmark

*Corresponding Author: [fbl@et.aau.dk](mailto:fbl@et.aau.dk)

***Note:*** As the supplementary file, one may find all the input data for the proposed novel correlation driven neural network. The Supplementary.zip comprises a folder named “Data” containing 450 samples (Data_Matrix_”No”.txt) with 24 feature candidates for each one and a value for the measured lifetime in hours. Following pattern shows the arrangement of the feature candidates for each sample. The source code of our proposed algorithm is embedded in the supplementary file. The codes are written in MATLAB. The main part of the source code, namely “CDNN_Main_File.m”, automatically imports these 450 samples, triggers the training process, and output the results. A detailed instruction of this source code is provided in the CDNN_Main_File.m file. Thus, one can run the CDNN_Main_File.m and see how the proposed method works.

| Hot dwelling temperature (^o^C) | Solder density(g/cm3) | Upper layer density(g/cm3) | lower layer density(g/cm3) |
| --- | --- | --- | --- |
| Hot dwelling time (min) | Solder CTE(10^-6^/ ^o^C) | Upper layer CTE(10^-6^/ ^o^C) | lower layer CTE(10^-6^/ ^o^C) |
| Heating ramp (^o^C /hour) | Solder Melting Temperature ( ^o^C) | Upper layer Melting Temperature ( ^o^C) | lower layer Melting Temperature ( ^o^C) |
| Cold dwelling temperature (^o^C) | Solder Young Module(GPa) | Upper layer Young Module(GPa) | lower layer Young Module(GPa) |
| Cold dwelling time (min) | Solder Poisson Ratio | Upper layer Poisson Ratio | lower layer Poisson Ratio |
| Cooling ramp (-^o^C /hour) | Solder Thickness(um) | Solder Width (mm) | Solder length (mm) |
| Measured lifetime (hours) | NA | NA | NA |
